# A Case of Papillary Growth from the Areola

**DOI:** 10.4103/0974-2077.69028

**Published:** 2010

**Authors:** Alok Vardhan Mathur, S Kudesia, M Anand, M Singh

**Affiliations:** *Department of Surgery, Shri Mahant Indiresh Hospital, Patel Nagar, Dehradun, India*; 1 *Department of Pathology, Shri Guru Ram Rai Institute of Medical Sciences, Patel Nagar, Dehradun, India*; 2 *Department of Surgery, Shri Guru Ram Rai Institute of Medical Sciences, Patel Nagar, Dehradun, India*

A 40-year-old female patient presented to the surgical clinic with complaints of a unilateral papillary growth arising from the areola since 30 years. There were no complaints of changes in size during pregnancy or lactation. General physical examination was unremarkable. Local examination showed a 1 cm × 1 cm pedunculated warty growth covered by skin, with a narrow stalk [Figure 1]. The lump was excised under local anaesthesia with a clinical diagnosis of papilloma. Histopathology is shown in Figure 2.

**Figure 1:**
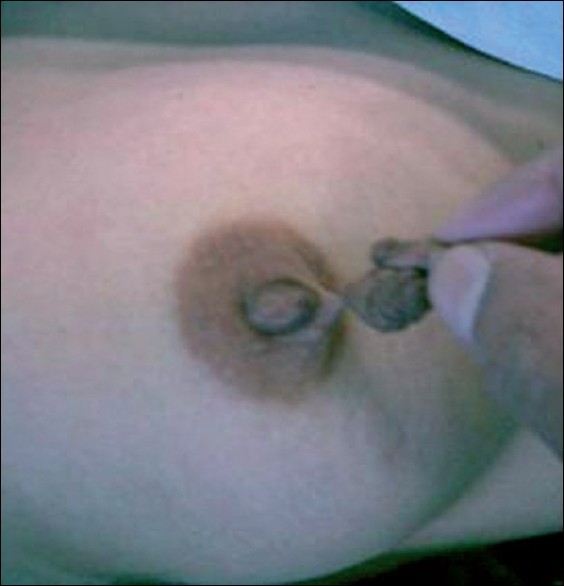
A papillary growth from areola with a narrow stalk

**Figure 2:**
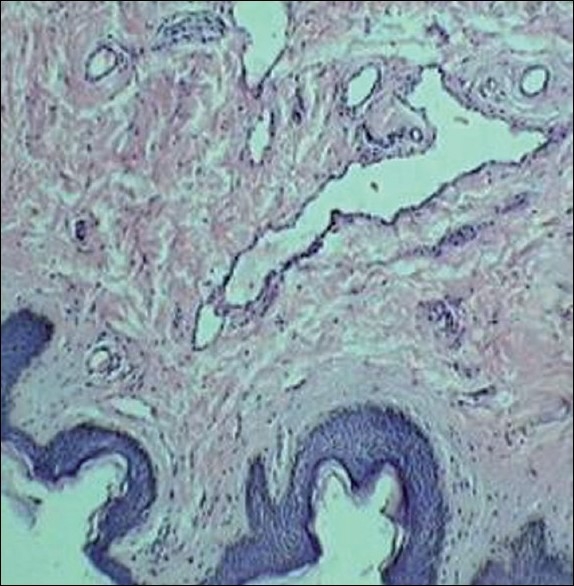
Histology of the papillary growth reveals ducts in dermis with normal overlying epidermis

What is your diagnosis?

## DIAGNOSIS:ACCESSORY NIPPLE

Histopathology showed ducts within stroma, confirming the diagnosis of accessory nipple. Supernumerary nipples (SNs), or polythelia, are located along the embryonic milk line. Ectopic SNs are found beyond the embryonic milk line.[1] SNs can appear complete with breast tissue and ducts, and are then referred to as polymastia, or they can appear partially with either of the tissues involved.

Nipple dichotomy, or intraareolar polythelia, involves one or more SNs within the same areola. These are very rare malformations. Their significance lies in their use for the reconstruction of a nipple defect resulting from surgery, e.g., a central quadrantectomy.[2]

They result from failure of regression of the ectodermal streaks formed along the primitive mammary line. Hence, they are located along the milk line, although ectopic nipples have been reported from as far as the toes.

The first SN was reported as early as in 1878. The incidence is reported at 0.2%.[3] There is a male preponderance, with a male to female ratio of as high as 1.7:1. In the last two decades, there has been renewed interest in SNs because of the dilemma of the possible associated malformations.

## ASSOCIATED ABNORMALITIES

SN has been described in a number of syndromes but, in most cases, they are probably a chance finding. These syndromes include Turner syndrome, Fanconi anaemia, haematologic disorders[4] and ectodermal dysplasia.

Other associations of SNs include the following;

Central nervous system anomalies like aneurysms, epilepsy, migraine, neurosis and neural tube defects, gastrointestinal anomalies like acid pepsin disease and pyloric stenosis, skeletal malformations of the hand, vertebra, coronal suture synostosis and rib absence, cardiac anomalies like bundle branch block, conduction anomalies and Patent ductus arteriosus (PDA) and renal anomalies.

There have been many studies to study the relationship between SN and these abnormalities, but none have been able to demonstrate a statistically significant association. Herein lies a medicolegal pitfall, and the possibility of litigation if such anomalies are not excluded, and, present later on, cannot be ignored.

## CLINICAL FEATURES

Clinically, they are often asymptomatic, or they may present with pain and swelling, associated with adolescence, pregnancy or menstruation. On examination, they are seen as small pigmented spots or as umbilicated nodules. They may be hidden by the normal breast. In the new born, for the detection of SN, it is useful to press a wet gauze pad along the milk line, from the axilla to the thigh. When present, they are seen as concave areas of wrinkling. The ectopic sites are in the perineum, face, neck, shoulder and even the limbs!

Familial cases with autosomal-dominant transmission with incomplete expression are also known to run in a family.[5]

## CLASSIFICATION

They were classified on the basis of the presence of glandular tissue, nipple, areola, fat and hair, by Kajava in 1915, as follows:[6]

Type I: Glandular tissue with nipple, areola and fat
Type II: Glandular tissue with nipple, no areola
Type III: Glandular tissue with areola, no nipple
Type IV: Glandular tissue only
Type V: Pseudomamma – nipple, areola present, no glandular tissue
Type VI: Polythelia – only nipple present
Type VII: Polythelia areolaris – only areola present
Type VIII: Polythelia pilosa – hair patch present

Our case of intraareolar polythelia had ductal tissue, stroma and skin, evident only on histology, whereas on gross examination, only a papillary growth from the nipple was evident. Hence, it is distinct from Kajava’s classification.

## DIFFERENTIAL DIAGNOSIS

They need to be differentiated from pigmented naevi, neurofibroma, dermatofibroma, hidradenitis supppurativa, lipoma and other skin tags. However, atypical cases like the one presented above may only be diagnosed on histology findings of areolar tubercles, smooth muscle of the areola, mammary glands and intradermal ducts.[7] SN may be a precursor of extra-mammary Paget’s disease[8]

## CLINICAL CLUE TO DIAGNOSIS

Clinically, the presence of wrinkling on folding between the fingers or the presence of a cleft-like appearance in the centre should make one suspect the condition. On dermoscopy, these lesions show central white scar-like areas with peripheral fine pigmentation.

## TREATMENT

Treatment objectives should be:

1. To establish the diagnosis and offer cosmetic improvement if required. Some patients with small flat nipples may need only follow-up. Others with protuberant erectile growths may need simple surgical excision. Removal using liquid nitrogen has been described.[9] The tumescent liposuction is another technique that offers more cosmetic results.[10]
2. Work-up to rule out an associated congenital malformation. Renal abnormalities are the most common and should be excluded by means of ultrasonography. However, on review of the literature, although there are conflicting results in various series, the relationship has not been conclusively established.
3. Exclusion of pathology in the SN itself or the associated malformation. Fine needle aspiration cytology, mammography, ultrasound and magnetic resonance imaging may be performed to rule out secondary changes. Women with a family history of breast cancer may merit excision of the nipple along with any associated breast tissue.
